# Isolated Adrenocorticotropic Hormone (ACTH) Deficiency as an Immune-Related Adverse Event Following Combination Immune Checkpoint Inhibitor Therapy

**DOI:** 10.7759/cureus.62863

**Published:** 2024-06-21

**Authors:** Mari Matsushiro, Kimitaka Shibue, Kazuki Osawa, Akihiro Hamasaki

**Affiliations:** 1 Department of Diabetes and Endocrinology, Medical Research Institute Kitano Hospital, PIIF Tazuke-Kofukai, Osaka, JPN; 2 Department of Gastroenterology, Medical Research Institute Kitano Hospital, PIIF Tazuke-Kofukai, Osaka, JPN

**Keywords:** acth deficiency, low cortisol, pituitary hormones, immune checkpoint inhibitors, immune-related adverse event

## Abstract

Isolated adrenocorticotropic hormone (ACTH) deficiency is a rare condition characterized by the sole impairment of ACTH secretion among the various hormones produced by the pituitary gland. This leads to secondary hypoadrenocorticism, manifesting symptoms such as fatigue, anorexia, weight loss, and altered consciousness. Recently isolated ACTH deficiency has emerged as an immune-related adverse event (irAE) associated with immune checkpoint inhibitors (ICIs). In this report, we detail a case of isolated ACTH deficiency as a result of irAE. A 65-year-old man received nivolumab and ipilimumab combination therapy for esophageal cancer and approximately six weeks later, presented fatigue and anorexia, and was shown hyponatremia and hyperkalemia on blood test, and was diagnosed as isolated ACTH deficiency. Retrospective examination indicated an increase in eosinophils and a slight decrease in sodium levels shortly before thyrotoxicosis was diagnosed. These findings suggest the possibility of mild hypoadrenocorticism, potentially due to decreased ACTH secretion, existing prior to the recognition of adrenal insufficiency symptoms. Healthcare providers should maintain a heightened vigilance for eosinophilia and electrolyte imbalances during the administration of ICIs. The detection of even subtle abnormalities in these parameters should prompt immediate consultation with an endocrinologist.

## Introduction

Isolated adrenocorticotropic hormone (ACTH) deficiency is a rare condition characterized by the sole impairment of ACTH secretion among the various hormones produced by the pituitary gland. This leads to secondary hypoadrenocorticism, manifesting symptoms such as fatigue, anorexia, weight loss, and altered consciousness. While more prevalent in adult males, typically presenting in their 50s, its incidence remains low. As a point of reference, the prevalence of acquired isolated ACTH deficiency was reported to be 3.8 to 7.3 per 100,000 people [1]. Interestingly, isolated ACTH deficiency has emerged as an immune-related adverse event (irAE) associated with immune checkpoint inhibitors.

While irAEs can affect multiple organs, presenting as colitis, hepatitis, interstitial pneumonia, dermatitis, neuromuscular disorders, and various endocrine disorders [2], endocrine manifestations encompass hypopituitarism, hypoadrenocorticism, hyperthyroidism, hypoparathyroidism, and type 1 diabetes mellitus [3]. Notably, combined therapy using ipilimumab (an anti-cytotoxic T-lymphocyte associated protein 4 (CTLA-4) antibody) and nivolumab (an anti-programmed cell death protein 1 (PD-1) antibody) has shown pituitary dysfunction in 7.68-10.5% of cases [4] with isolated ACTH deficiency being the most prevalent.

In this report, we detail a case of isolated ACTH deficiency as a result of irAE.

## Case presentation

A 65-year-old man presented to our hospital with a primary complaint of hoarseness and was diagnosed with Stage IV esophageal cancer. His medical history included hypertension, hyperuricemia, and cirrhosis of the liver. He had a smoking habit of 15 cigarettes per day and a drinking habit of about 750 ml of beer. Family history included prostate cancer in the father and thyroid disease in the mother, details of which were unknown.

He commenced treatment with nivolumab and ipilimumab, both immune checkpoint inhibitors (ICIs). A blood test conducted four weeks later indicated a decreased thyroid-stimulating hormone (TSH) level of 0.035 μIU/mL (0.035 mU/mL). Consequently, he was monitored for destructive thyroiditis attributed to irAE. About 10 weeks after the start of ICIs, he developed fatigue and anorexia. Five days after that, during a routine outpatient blood test, hyponatremia and hyperkalemia were observed. Since he was undergoing treatment with an immune checkpoint inhibitor, irAE was suspected. The afternoon blood test performed at that time showed relatively low levels of ACTH, 4.32 pg/mL (0.95 pmol/L) and cortisol, 2.1 μg/dL (57.9 nmol/L), but his general condition was relatively good and he was able to walk to the hospital unassisted.

**Figure 1:**
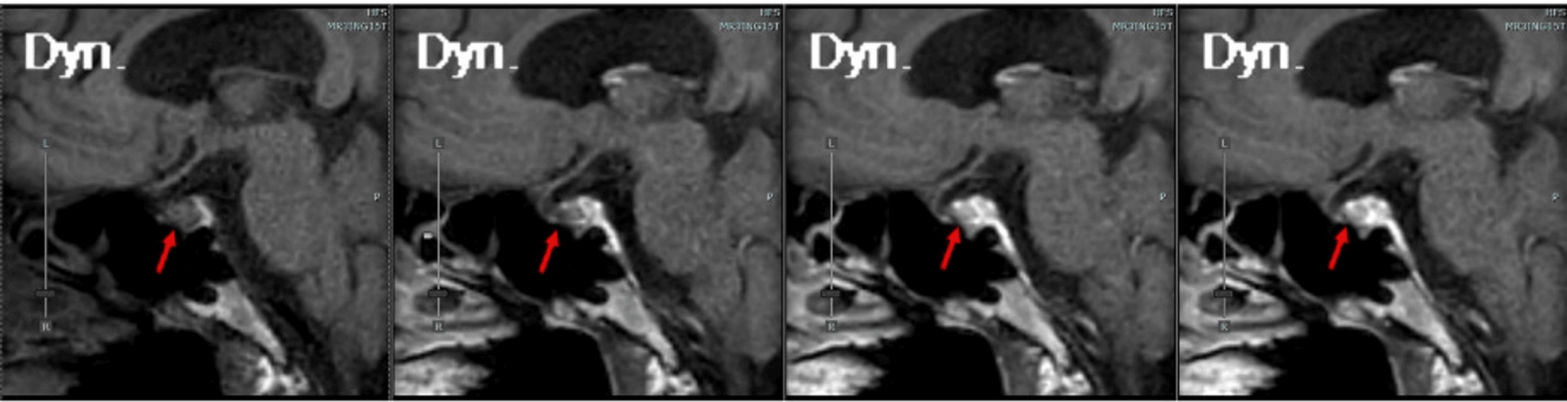
Sagittal view of contrast-enhanced pituitary MRI. Sagittal view of contrast-enhanced pituitary MRI, progressing from early phase to late phase, from left to right. The red arrow points to the pituitary gland, and there are no obvious abnormalities.

Seven days after the onset of the symptoms, after 30 minutes of early morning fasting and bed rest, blood tests revealed low ACTH, <1.5 pg/mL (<0.33 pmol/L) and cortisol, 1.4 μg/dL (38.6 nmol/L), as well as progressive hyponatremia. He exhibited generalized fatigue and anorexia, with a blood pressure reading of 91/55 mmHg. However, there were no physical signs indicative of overt adrenal insufficiency, such as joint pain. Contrast-enhanced pituitary MRI showed no abnormalities in the pituitary gland or pituitary pattern (Figure 1).

**Figure 2:**
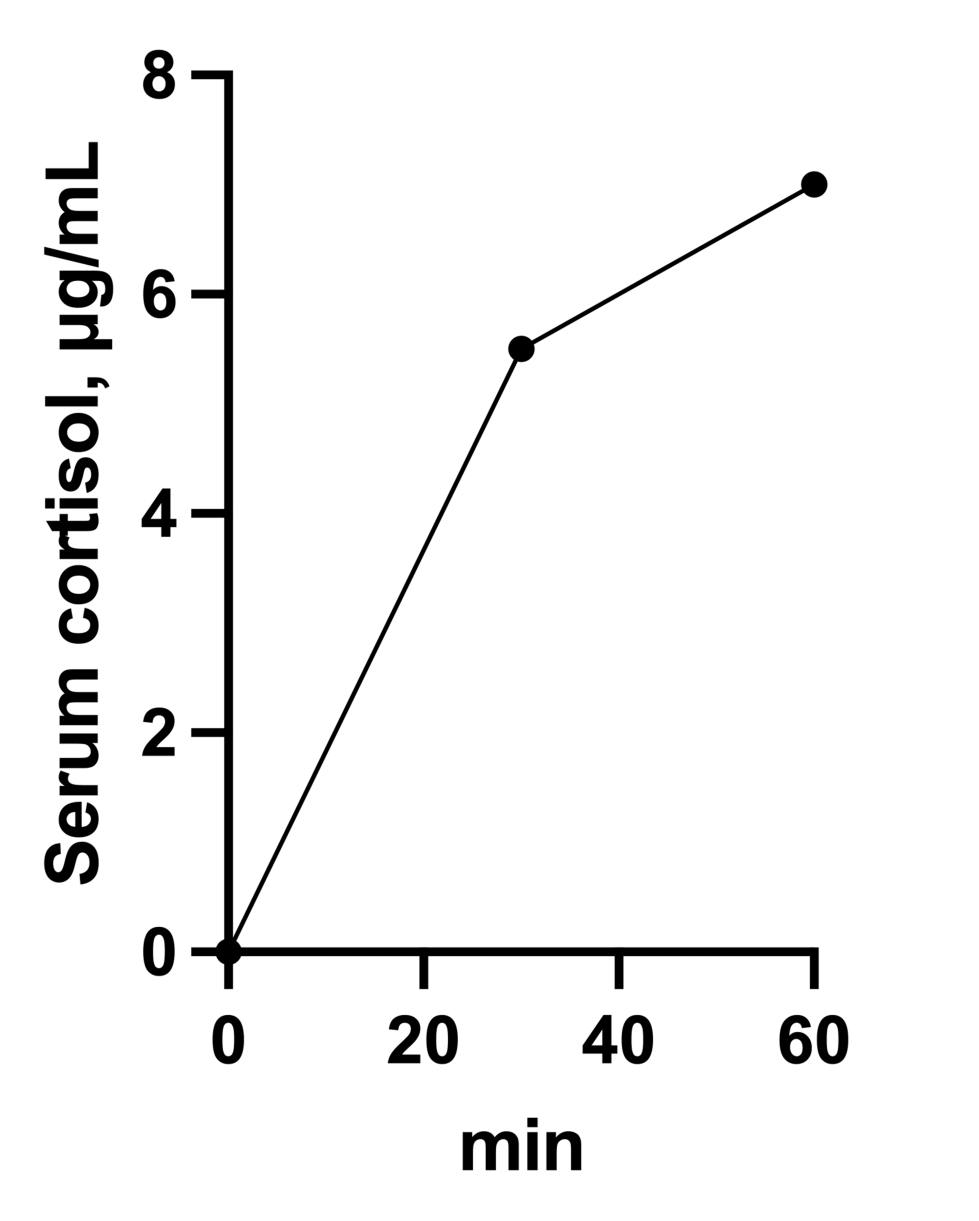
Results of the rapid adrenocorticotropic hormone (ACTH) challenge test. This graph represents the results of the rapid ACTH challenge test. The peak cortisol value is 7 μg/mL, suggesting adrenal insufficiency.

Blood tests (Table 1) showed mildly low levels of insulin like growth factor 1 (IGF1), which was considered to be influenced by cirrhosis. The gonadal system was also considered consistent with age-related changes. A rapid ACTH challenge test (Figure 2) showed low cortisol response but a response to ACTH. In the corticotropin-releasing hormone (CRH) challenge test (Table 2), both ACTH and cortisol consistently remained below the detection threshold, and there was no response observed for either hormone. Based on the above, we diagnosed the patient as having an isolated ACTH deficiency associated with irAE, since there were no pituitary hormonal abnormalities other than ACTH and cortisol, no pituitary abnormalities on imaging, and a history of three months after the use of immune checkpoint inhibitors.

**Table 1:** Laboratory investigations at presentation.

		The reference range
Plasma adrenocorticotropic hormone, pg/mL	<1.5	7.2-63.3
Serum cortisol, μg/mL	1.4	7.07-19.6
Serum growth hormone, ng/mL	1.34	≦2.47
Serum insulin-like growth factor-1, ng/mL	44	72-221
Serum thyrotropin, μIU/mL	4.241	0.61-4.23
Serum free triiodothyronine, pg/mL	2.44	2.52-4.06
Serum free thyroxine, ng/dL	0.94	0.75-1.45
Serum luteinizing hormone, mIU/mL	6.21	0.79-5.72
Serum follicle-stimulating hormone, mIU/mL	8.38	2.00-8.30
Serum free testosterone, pg/mL	4.0	5.3-11.5
Serum prolactin, ng/mL	9.17	4.29-13.69
Plasma antidiuretic hormone, pg/mL	0.6	≦2.8
Serum aldosterone, pg/mL	27.7	4.0-82.1
Plasma renin activity, ng/mL/hr	16.1	0.2-2.3
Serum dehydroepiandrosterone sulphate, μg/dL	14	24-244
Anti-thyroid peroxidase antibody, IU/mL	<9.00	<3.3
Anti-thyroglobulin antibody, IU/mL	123.00	<28

**Table 2:** Results of the corticotropin-releasing hormone (CRH) challenge test.

	0min.	30min.	60min.	90min.	120min.
Plasma adrenocorticotropic hormone, pg/mL	<1.50	<1.50	<1.50	<1.50	<1.50
Serum cortisol, μg/mL	<1.0	<1.0	<1.0	<1.0	<1.0

Since the patient was hypotensive and febrile, hydrocortisone 100 mg was administered intravenously on the first day. We then confirmed that there were no symptoms, such as daytime malaise (a feeling of general discomfort or uneasiness during the day), or any other issues arising from the hydrocortisone supplementation of 15 mg/day as a physiologic dose of cortisol replacement. On sick days, patients were instructed to take three times the usual dose, amounting to 45 mg of hydrocortisone orally.

The patient was discharged with a maintenance regimen of hydrocortisone at a dosage of 15 mg daily. Subsequent follow-ups have revealed no clinical or biochemical signs of adrenal insufficiency, such as fatigue, anorexia, or electrolyte imbalances.

## Discussion

In this report, we present a case of ACTH deficiency caused by ICI administration, which was accompanied by preceding destructive thyrotoxicosis and successfully treated with cortisol supplementation.

Under the use of anti-PD-1 antibodies, the incidence of hypophysitis is rare, occurring in less than 1%. However, with anti-CTLA-4 antibodies, it occurs in 10% [5]. Furthermore, when both therapies are combined, hypophysitis is reported to occur at approximately twice the frequency of anti-CTLA-4 antibody monotherapy [4]. This case involved combined therapy with anti-PD-1 antibodies and anti-CTLA-4 antibodies, carrying a high risk of hypophysitis.

Thyrotoxicosis caused by irAE often occurs two to six weeks after the start of ICI [6]. In this case, destructive thyroiditis developed approximately four weeks after the start of ICI and the symptoms of ACTH deficiency caused by irAE were prominent at about 10 weeks after the start of ICI, which was consistent with the predominant period of pituitary inflammation caused by irAE, which is around 10 weeks [7]. There have been several reports of cases with concurrent thyroid dysfunction and isolated ACTH deficiency, as in this case [8]. In all cases with coexistence of both conditions, thyroid dysfunction either preceded or occurred simultaneously with isolated ACTH deficiency, and no cases were found where isolated ACTH deficiency preceded thyroid dysfunction. In this case, as in previous reports, the patient developed destructive thyroiditis before isolated ACTH deficiency.

In this case, retrospective examination indicated an increase in eosinophils and a slight decrease in sodium levels shortly before thyrotoxicosis was diagnosed (Table 3).

**Table 3:** Time series of test values. ICI: immune checkpoint inhibitors

	reference range	the day before ICIs	2 weeks later	4 weeks later	6 weeks later	8 weeks later	11 weeks later
plasma glucose, mg/dL	73-109	128	152	177	116	132	97
serum sodium, mEq/L	138-145	139	137	136	133	140	122
serum potassium, mEq/L	3.6-4.8	4.4	4	4.5	4.8	4.0	5.3
serum chloride, mEq/L	101-108	104	102	99	99	105	92
corrected serum calcium, mg/dL	8.8-10.1	9.6	9.9	9.8	10.0	9.6	9.7
serum creatinine, mg/dL	0.65-1.07	0.88	0.88	0.93	0.84	0.86	1.08
white blood cell, /μL	3300-8600	5600	5200	5100	6500	6600	4600
neutrophils, %	41.7-73.7	72.6	73.7	72.6	62.2	70.8	61.1
lymphocytes, %	18.4-44.8	22	17.6	16.7	17.4	18.7	21.4
eosinophils, %	0.7-8.1	0.7	2.3	3.9	9.5	5.0	9.9
Serum thyrotropin, μIU/mL	0.61-4.23	1.303	0.138	0.035	0.009		4.241
Serum free triiodothyronine, pg/mL	1.68-3.67	2.07	3.12		3.08		2.44
Serum free thyroxine, ng/dL	0.70-1.48	0.85	1.12	1.36	1.68		0.94
Plasma adrenocorticotropic hormone, pg/mL	7.2-63.3	21.30	19.30				<1.50
Serum cortisol, μg/mL	3.7-19.4	8.5	9.3				1.4

These findings suggest the possibility of mild hypoadrenocorticism, potentially due to decreased ACTH secretion, existing prior to the recognition of adrenal insufficiency symptoms. Furthermore, the development of symptoms of an upper respiratory tract infection a few days before the adrenal insufficiency symptoms appeared might have triggered an increased demand for cortisol, leading to the manifestation of clinical signs. There is a report that suggests the possibility of predicting the occurrence of isolated ACTH deficiency by the decrease of free thyroxine that starts 12 weeks before the decline of cortisol secretion [9]. Considering these elements collectively, it is plausible that in cases of concurrent thyroid dysfunction and isolated ACTH deficiency due to irAEs, both thyroid and pituitary abnormalities are a consequence of a common mechanistic pathway affecting the endocrine glands. The divergence in their clinical manifestation is primarily a factor of timing. Further, an additional report documents a scenario in which adrenal insufficiency was manifested and isolated ACTH deficiency was diagnosed subsequent to levothyroxine supplementation for thyroid dysfunction precipitated by irAEs [10].

In light of the fact that hyponatremia and eosinophilia are known to be the main laboratory abnormalities in isolated ACTH deficiency due to irAE [10], it becomes imperative for healthcare providers to maintain a heightened vigilance for eosinophilia and electrolyte imbalances during the administration of ICIs. Thyroid hormones accelerate the metabolism of cortisol, which may manifest latent adrenal insufficiency in hyperthyroidism or under levothyroxine treatment. Therefore, special attention should be paid to the changes in laboratory values such as eosinophilia and hyponatremia when patients develop thyrotoxicosis or hypothyroidism due to irAEs and receive levothyroxine replacement. The detection of even subtle abnormalities in these parameters should prompt immediate consultation with an endocrinologist. Then it will be required to conduct a comprehensive adrenal function test, such as a loading test, to assess the integrity of the hypothalamic-pituitary-adrenal (HPA) axis.

Furthermore, given the potential for adrenal insufficiency, particularly in stressful physiological states, the need for cortisol supplementation during periods of illness ('sick day rules') should be carefully considered. This approach not only aids in timely identification and management of adrenal insufficiency but also ensures the optimization of overall patient care, especially in the context of complex oncological treatments involving ICIs. Such proactive measures are vital for mitigating potential endocrine complications, thereby enhancing the safety and efficacy of ICI therapy.

## Conclusions

This case highlights the importance of recognizing and managing ICI-induced endocrine disorders, particularly isolated ACTH deficiency, which often occurs after thyroid dysfunction. In this case, retrospective examination indicated slight changes in laboratory data shortly before thyrotoxicosis was diagnosed. In other words, before the symptoms of adrenal insufficiency appeared, changes in test results had occurred. A proactive approach to monitoring laboratory abnormalities such as eosinophilia and hyponatremia is crucial, as they are early indicators of ACTH deficiency. Healthcare professionals should ensure comprehensive adrenal function testing and maintain vigilance during stressful physiological states, optimizing the management of ICI therapy. This approach enhances patient safety, emphasizing the critical need for collaboration with endocrinologists for early detection and treatment.
